# A Large-Scale Analysis of Corrosion Data in High-Sulfur Natural Gas Purification Systems

**DOI:** 10.1021/acsomega.6c03934

**Published:** 2026-08-06

**Authors:** Qinghua Meng, Wenjie Zhang, Lianggui Liu, Kai Chen, Chunlin Xiang, Ya Deng, Cheng Zhong

**Affiliations:** † Sinopec Guangyuan Natural Gas Purification Co., Ltd, Guangyuan 628017, China; ‡ College of Chemistry and Chemical Engineering, 74602 Southwest Petroleum University, Sichuan 610500, China; § State Key Laboratory of Oil and Gas Reservoir Geology and Exploitation, Southwest Petroleum University, Sichuan 610500, China

## Abstract

Corrosion is a significant environmental and safety concern of high-sour natural gas purification systems. However, complex pipeline configurations and corrosion mechanisms frequently lead to ineffective corrosion prevention and control in high-sulfur purification systems. To address this, artificial intelligence and data-driven approaches show significant promise. However, their practical applicability in large industrial purification systems remains insufficiently explored. In this study, a large-scale corrosion database containing 107,255 inspection records collected from 4,258 monitoring locations between 2018 and 2024 was established for a high-sulfur natural gas purification plant in China. A database incorporating time-series machine learning models was established for the rapid analysis and modeling of large corrosion data sets. The results indicate that inspection records are unevenly distributed across pipeline loops and component types, with monitoring data mainly concentrated in several key amine circulation pipelines. Most inspection points are located on straight pipes and elbows, while tees and reducers account for a relatively small proportion. A total of 21,463 valid time-series records were identified for forecasting analysis. Among the evaluated forecasting approaches, the ETS–Theta hybrid model exhibited the broadest applicability and achieved stable predictions in 94% of eligible monitoring locations. The model successfully identified high-corrosion-risk areas such as acid gas (AG), acid water (AW), and low-pressure condensate (LC). The prediction results were further validated through time-series cross-validation, semiempirical corrosion models, field investigations, and corrosion-product characterization. This study provides new insights into the actual corrosion data and the high-risk zones indicated by these data within high-sulfur gas field purification systems. Furthermore, the proposed framework establishes a practical basis for large-scale corrosion data governance, corrosion-risk identification, and intelligent corrosion management in high-sulfur natural gas purification systems.

## Graphical abstract

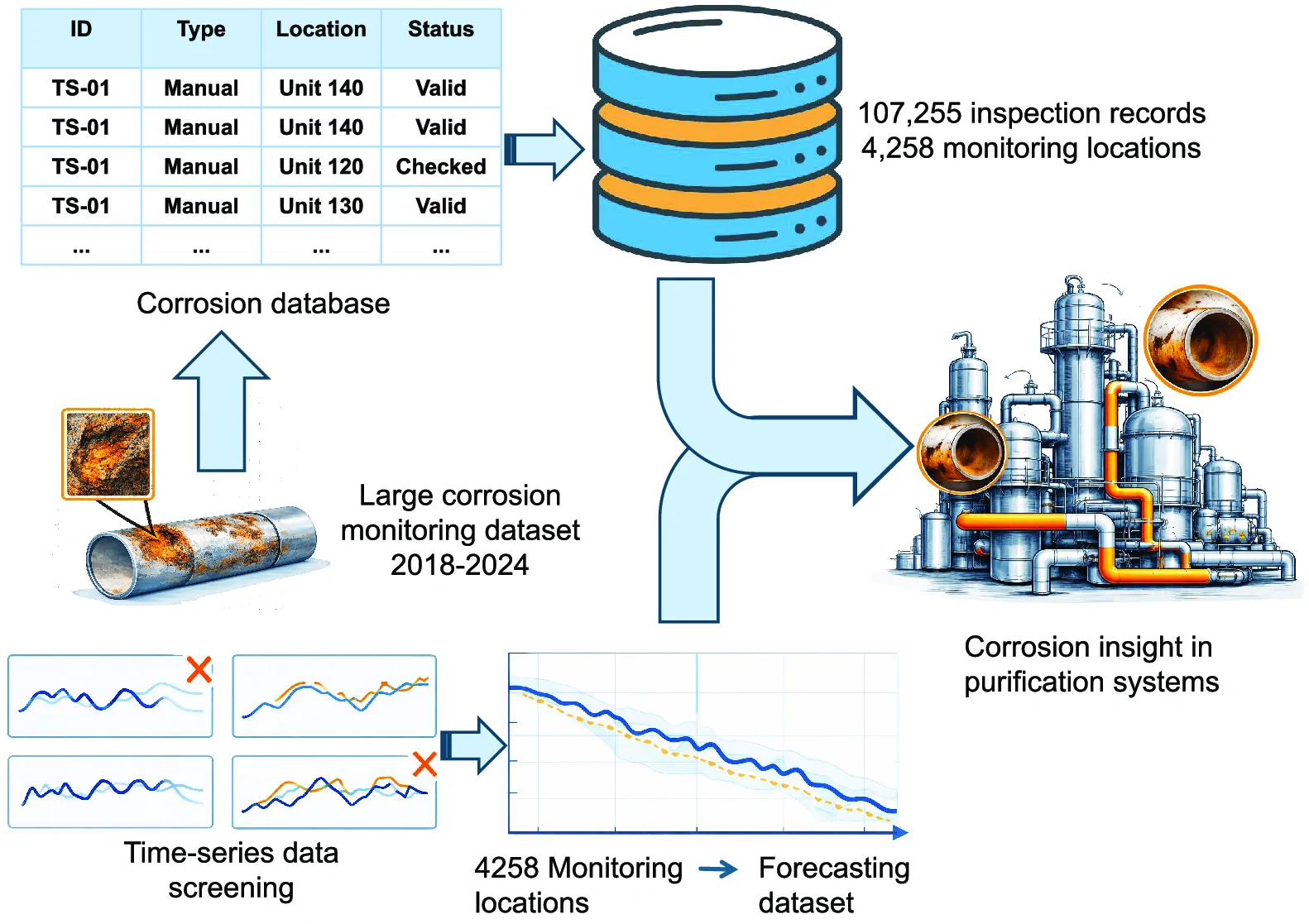

## 1 Introduction

Corrosion prevention and control in high-sulfur natural gas purification systems is a critical factor in ensuring the safe operation of gas processing plants.^1^ During long-term operation, purification systems are frequently exposed to severe corrosion caused by acidic media such as H_2_S. Under coupled conditions of high temperature, high pressure, and high sulfur content, corrosion behavior becomes highly complex and variable, which may lead to equipment perforation, medium leakage, and unplanned shutdowns. These issues often threaten the safe operation and life management of purification facilities.^2,3^

Corrosion monitoring data in purification plants are multisourced, variably formatted (including paper records), and dispersed across different departments and locations. These data commonly suffer from inconsistent formats, nonstandardized structures, and uneven quality, severely hindering data integration, systematic analysis and the development of predictive corrosion models.^4^ Although large amounts of corrosion monitoring data are available in industrial systems, corrosion rate estimation in practice still mainly relies on empirical or semiempirical models rather than data-driven approaches.^5^ Therefore, developing a proper understanding of the existing corrosion monitoring data and formulating effective strategies for their utilization has become an important research direction aimed at enhancing the safety management of purification systems.

Artificial intelligence techniques have been widely applied in corrosion prediction in recent years. Liu et al.^6^ proposed a support vector machine (SVM) model with a radial basis function kernel to predict internal corrosion rates in petroleum pipelines. Chern-Tong and Aziz^7^ developed the CMARGA model for corrosion prediction model for oil and gas pipelines, which integrates genetic algorithms with decision tree optimization, achieving an average prediction accuracy of 80.20% across multiple data sets. Ossai et al.^8^ applied a hybrid machine learning framework combining particle swarm optimization, neural networks, gradient boosting, and random forests to predict defect depth growth in aging pipelines, reducing the mean absolute percentage error (MAPE) to 6–8%. De Masi et al.^9^ demonstrated that integrating multiple information sources, such as pipeline geometry, fluid dynamics, and deterministic models, can improve corrosion risk identification. Their artificial neural network (ANN) ensemble model achieved *R* ^2^ values of 0.884 for corrosion rate and 0.672 for volume loss. Fang et al.^10^ applied RF and CatBoost algorithms to predict internal corrosion in heavy-oil pipelines using parameters including material composition, pressure, flow velocity, and temperature. Xie and colleagues developed a corrosion-rate prediction model for oil and gas pipelines based on a knowledge-graph-assisted neural network framework, achieving high predictive accuracy on real industrial data sets with coefficients of determination (*R* ^2^) exceeding 0.99.^11^ These studies demonstrate the strong potential of data-driven models for corrosion prediction. However, internal pipeline corrosion is governed by multiple coupled mechanisms, including electrochemical reactions, fluid flow conditions, and environmental variations, which introduce significant uncertainty into corrosion evolution and prediction.^12^ Although experimental data and simulated data are available, large-scale, long-term industrial field data have rarely been utilized.

This study analyzed over 100,000 corrosion monitoring records from a high-sulfur natural gas purification plant by developing a corrosion database and exploring prediction modeling. The goals were to evaluate the feasibility of data-driven prediction using field data and to identify previously underemphasized or not quantitatively defined corrosion monitoring weaknesses and high-risk zones.

## 2 Methods

### 2.1 Data Source and Data Preprocessing

Corrosion monitoring data were collected from a large high-sulfur natural gas purification plant in the Sichuan Basin. A total of 107,255 thickness measurement records were obtained from 2018 to 2024 at 4,258 fixed monitoring locations across four purification systems, supplemented by data from corrosion probes and online thickness monitoring devices. The monitoring points covered major corrosion-sensitive process units, including acid water stripping, liquid sulfur cooling, amine desulfurization, and dry gas separation. In addition to wall-thickness measurements, associated operational and record-level information, including pressure, flow velocity, temperature, pH, medium type, measurement point ID, equipment name, and inspection time, was collected where available. These data provide a reliable foundation for subsequent data cleaning, database construction, and machine learning model development.

### 2.2 Data Cleaning, Standardization, and Corrosion Database Development

To ensure data set completeness, accuracy, and consistency, systematic data acquisition, cleaning, and standardization were conducted (Figure 1). Subsequently, a corrosion data management platform was developed using Python v3.9 and a MySQL database. This platform integrated front-end and back-end modules for data storage, management, analysis, and visualization, enabling fast analyses and modeling of large-scale data sets.^13^

**Figure 1:**
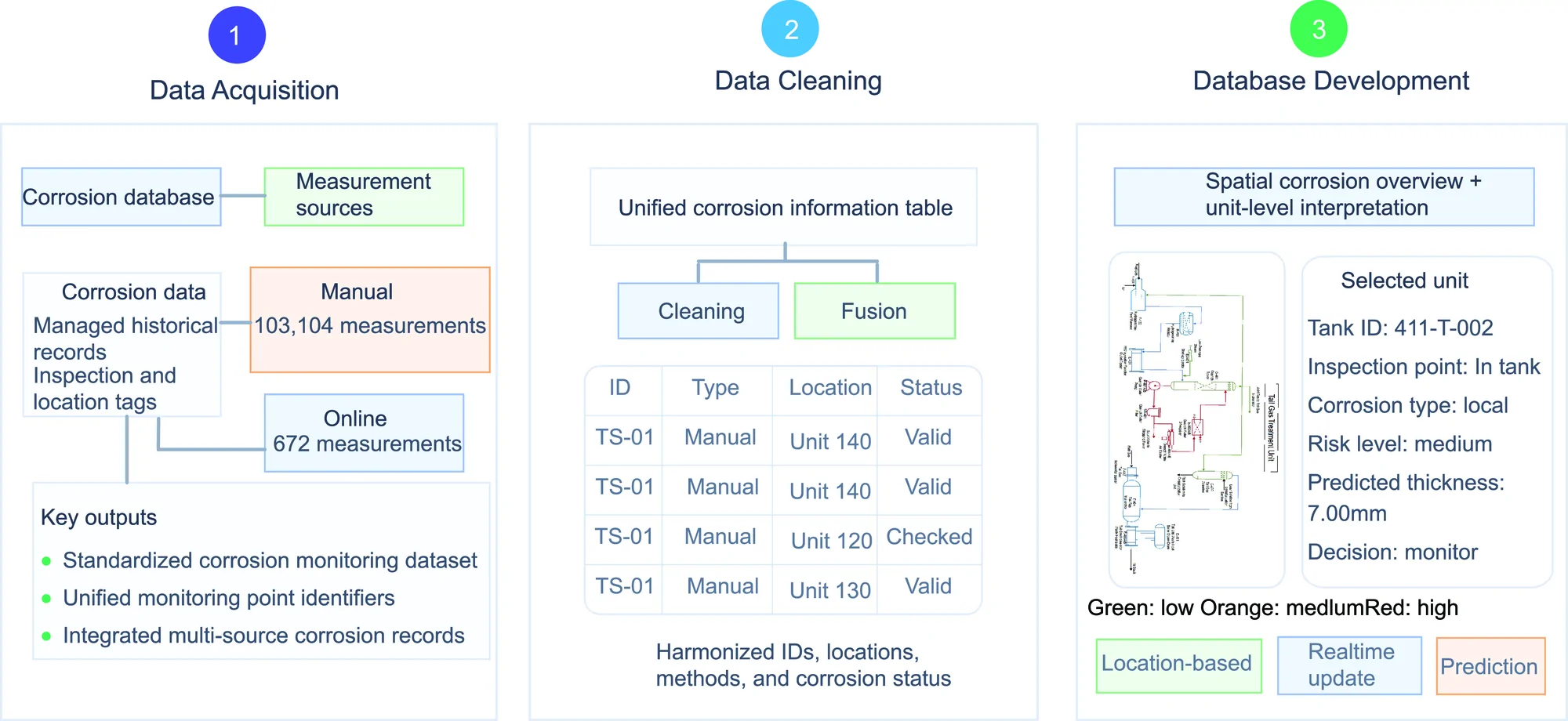
Workflow of corrosion data acquisition, integration, and visualization.

During the data sorting stage, the collected data were integrated into a unified corrosion information table through cleaning and data fusion. Abnormal data were identified, mainly including missing values, duplicate records, and format inconsistencies. For records with missing values or irregular formats, a forward-filling approach was adopted, in which valid thickness values from the previous year at the same monitoring point were used for correction. Records containing extreme anomalies or those that could not be corrected were removed. After the preliminary cleaning process, 3,342 noncompliant records were eliminated, accounting for approximately 3% of the total data sets.

Before time-series modeling, identifying and removing abnormal corrosion measurements is critical to ensure reliability. Given the non-normal distributions and irregular fluctuations of corrosion data, traditional methods like the 3σ rule or boxplot may fail to detect hidden outliers. Therefore, the Isolation Forest algorithm was introduced for deep anomaly detection.^14^

The Isolation Forest isolates anomalies by randomly partitioning the feature space and constructing multiple isolation trees. Points that are more easily isolated require fewer partitions and are considered anomalous. In this study, a model with 256 trees was built based on thickness variation statistics at each monitoring point, with a contamination parameter of 0.05. Samples predicted as abnormal were flagged as anomalies. Anomaly scores from the decision function were ranked, and samples with lower scores (more anomalous) were removed. As summarized in Table 1, 1,396 outliers were identified and removed from 328 monitoring points, mainly concentrated in liquid sulfur cooling and acid water stripping systems.

**Table 1:** Summary of Anomaly Detection Results Using the Isolation Forest Algorithm

data type	value
total records	22859
number of anomalous records	1396
total number of regions	4258
number of high-confidence anomalies	0
anomaly ratio	6.11%

To improve data consistency and enable cross-source integration, identifier standardization and equipment matching were performed as key steps in data organization (Figure 2). ID rule configuration and format recognition were first applied to uniformly parse equipment identifiers in the original inspection records. Different formats, including full, simplified, and extended, were converted into a unified standard format. Inspection report numbers were then extracted, and records were matched with specific pipeline equipment through Pipeline Equipment ID Mapping. For successfully matched identifiers, the corresponding monitoring point part numbers were identified, and thickness data were recorded accordingly. Failed matches underwent manual processing and verification. After identifier standardization, equipment mapping, and thickness data entry, the results were exported and reviewed to generate standardized corrosion monitoring records.

**Figure 2:**
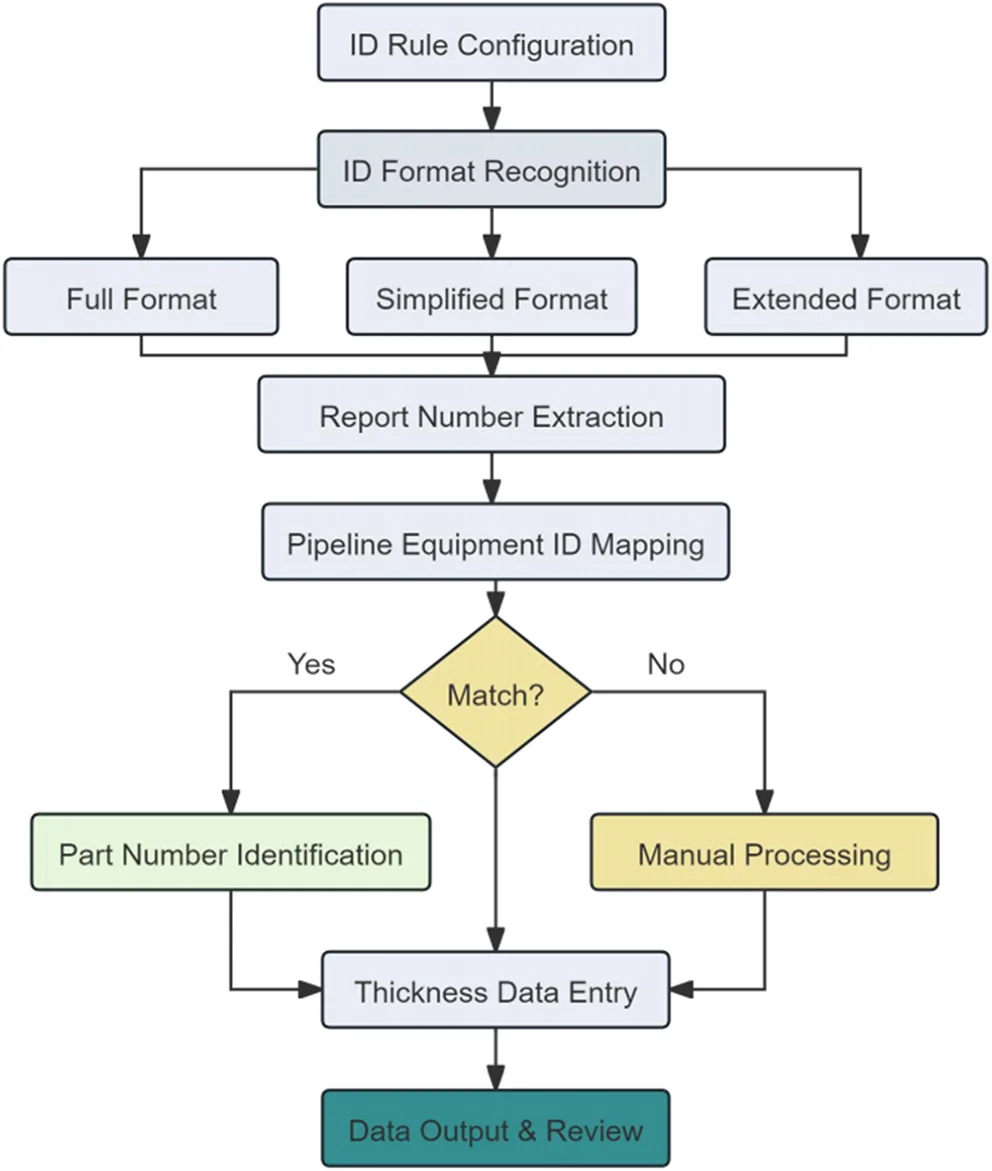
Workflow of thickness data standardization and pipeline equipment ID mapping.

Based on the cleaned and standardized data, a unified corrosion information table was established and further organized into standard time-series data sets in the format of “Monitoring Point ID-Year-Thickness.” Consequently, 4,258 valid monitoring points and 21,463 time-series records were obtained, which exhibited stable structure and clear temporal trends, providing a reliable data foundation for subsequent corrosion prediction modeling using the ETS-Theta, ARIMA, SVR, and LSTM models.

All analyses were performed in Python 3.9. The Isolation Forest algorithm described in Section 4 was implemented using scikit-learn (v1.5.0),^15^ whereas ETS and Theta forecasting models were implemented using statsmodels (v0.14.2).^16^ The ETS model was configured as a nonseasonal additive trend model without seasonal components, and the classical nonseasonal Theta formulation was adopted for the Theta model. The hybrid ETS–Theta model combined predictions from the two models using the *R* ^2^-weighted averaging strategy described in Equation in eq 7.

### 2.3 Large-Scale Analyses of Corrosion Data

Large-scale data analyses were conducted to characterize the overall structure of the corrosion monitoring data sets. The temporal distribution of monitoring data was analyzed by summarizing the annual number of inspection records to evaluate inspection frequency and data availability. The spatial distribution of monitoring points was analyzed across different process units, pipeline loops, and structural components to assess monitoring coverage throughout the purification plant. Additionally, the proportions of different monitoring methods, including manual ultrasonic thickness measurements, corrosion probes, and online monitoring systems, were calculated to evaluate the composition of the data sets. The distribution of monitoring locations among different structural components, including straight pipes, elbows, tees, and reducers, was also statistically summarized.

### 2.4 Time-Series Models

The preliminary data analyses suggest that the overall characteristics of the time series, the data showed a structure dominated by long-term trends with superimposed random fluctuations, without a stable seasonal pattern. Therefore, nonseasonal forms of the ETS and Theta models were adopted for modeling to ensure consistency between the model assumptions and the underlying corrosion mechanisms.^17,18^

The ETS model was expressed as follows

1 $$l_{i}=ay_{i}+\left(1-a\right){\left(l_{i+1}+b_{i+1}\right)}_{e}$$

2 $$b_{i}=\beta \left(l_{i+1}-l_{i}\right)+\left(1-\beta \right)b_{{i+1}_{e}}$$

3 $${ŷ}_{i+k}=l_{i}+kb_{i_{e}}$$

where l_i_ represents the current level component of the time series; b_i_ denotes the long-term trend component; y_i_ is the observed value of the time series; a is the level smoothing parameter used to smooth the changes in the level component; β is the trend smoothing parameter used to smooth the changes in the trend component; and ŷ_i+k_ represents the k-step-ahead forecast value.

The Theta model can be expressed as

4 $$y_{t}=\theta_{t}+\varepsilon_{t}$$

5 $$\theta_{t}=\alpha y_{t}+\left(1-\alpha \right)\theta_{t-1}$$

6 $${ŷ}_{t+h}=\theta_{t}+h\cdot b$$

where y_t_ denotes the observed value of the original time series; θ_t_ represents the trend component (typically obtained through exponential smoothing); α is the smoothing parameter used to control the update rate of the trend; b represents the trend estimate; and h denotes the prediction step.

A single time-series forecasting model has inherent limitations and may not fully capture complex temporal patterns or future variation trends. Consequently, ensemble or hybrid modeling approaches are often adopted to improve forecasting accuracy and robustness.^19^ Therefore, based on the predictions obtained from individual models, a weighted average ensemble approach was adopted to combine the advantages of both models. The ETS and Theta models were first used separately to perform time-series prediction, producing two sets of forecast results. The coefficients of determination *R* ^2^ for the ETS and Theta models were 0.76 and 0.58, respectively. Subsequently, a weighted average of the two model outputs was calculated. The resulting weighting coefficient α was 0.569, forming the ETS–Theta hybrid prediction model.

Using this hybrid model, corrosion trends from 2025 to 2030 were predicted. According to the requirements specified in TSG D7005—2018, if the predicted corrosion depth of a pipeline exceeds 30% of the wall thickness, the corresponding pipeline section should be replaced.^20^Therefore, a 30% threshold of wall thickness loss was adopted as the warning criterion for corrosion risk in this study.

The model fusion is performed according to the following equation

7 $${ŷ}_{\mathrm{final}}=\alpha {ŷ}_{i+k}+\left(1-\alpha \right){ŷ}_{t+h}$$

where ŷ_final_ is the final predicted value, and α is the weighting coefficient.

The coefficient of determination *R* ^2^ was used to evaluate the overall predictive performance of the ETS and Theta models on the field data sets

8 $$R^{2}=1-\frac{\sum {\left(y-ŷ\right)}^{2}}{\sum {\left(y-\mathrm{y̅}\right)}^{2}}$$

where y represents the actual value, ŷ represents the predicted value, and y̅ denotes the mean of the observed values.

Based on the *R* ^2^ values of the ETS and Theta models, the weighting coefficient α was determined to construct the ETS–Theta hybrid model.

9 $$\alpha =\frac{R_{\mathrm{ETS}}^{2}}{R_{\mathrm{ETS}}^{2}+R_{\mathrm{Theta}}^{2}}$$

Consequently, a greater weight was assigned to the model exhibiting superior forecasting accuracy when constructing the ETS–Theta hybrid model.

### 2.5 Model Comparison and Validation

To evaluate the suitability of time-series models for corrosion prediction using large-scale field data, this study selected the Exponential Smoothing–Theta (ETS-Theta) model as the primary approach. Its performance was benchmarked against ARIMA, SVR, and LSTM under consistent settings. Model performance was assessed using MAE, sMAPE, and *R* ^2^. Beyond quantitative metrics, robustness and practical reliability were evaluated through time-series cross-validation, field corrosion investigation, and comparisons with semiempirical models. To further verify the corrosion conditions indicated by the modeling results, on-site investigations were conducted in key process units, focusing on high-risk areas such as acid gas pipelines, acid water systems, and low-pressure condensate units.

#### 2.5.1 Validation Using Semi-Empirical Model Fitting

Machine learning models are often regarded as “black-box” models due to their limited interpretability in explaining the underlying mechanisms of predictions.^21^ In this study, two commonly used semiempirical models, the Norsok model and the DWM model, were semiempirical to cross-validate the time-series prediction results. These semiempirical models estimate corrosion rates using key environmental and operational parameters. For example, the De Waard–Milliams (DWM) model predicts CO_2_ corrosion rates primarily as a function of temperature and CO_2_ partial pressure, while later modifications incorporated additional parameters such as pH, water chemistry, pressure, and fluid flow velocity to better represent field environments.^22^ Similarly, the Norsok model estimates corrosion rates using parameters including temperature, pH, CO_2_ fugacity (or partial pressure), and wall shear stress associated with fluid flow conditions.^23^

The Norsok model is expressed as

10 $$\mathrm{CR}=A\cdot e^{B\cdot T\cdot V^{C}}$$

where CR is the corrosion rate; A, B, and C are constant coefficients; T represents temperature (°C); and V denotes the flow velocity (m/s).

The DWM model is expressed as

11 $$V_{c}=A\cdot M_{H_{2}S}\cdot \exp\left(\frac{-Q}{\mathrm{RT}}\right)$$

where V_c_ is the corrosion rate (mm/year); A is a constant coefficient; M_H_2_S_ represents the concentration of hydrogen sulfide gas (mg/m^3^); *T* denotes temperature (°C); *Q* is the activation energy (kJ/mol); and *R* is the gas constant.

#### 2.5.2 Time Series Cross-Validation

Model stability and reliability were also assessed across 269 eligible locations using a rolling window strategy with a 24-month training set and a 6-month test set.^24^

Assuming the time-series length is *n*, the training window size is *w*, and the testing window size is *m*, the total number of validation iterations in TSCV can be expressed as

12 $$k=\left\lfloor \frac{n-w}{m}\right\rfloor$$

The loss function for each validation step is defined as

13 $$L_{i}=\frac{1}{m}\sum_{t=w+\left(i-1\right)m+1}^{w+\mathrm{im}}\left|y_{t}-{ŷ}_{t}\right|$$

where L_i_ represents the loss value in the i validation iteration, y_t_ denotes the actual value at time t, ŷ_t_ represents the predicted value at time t, and i indicates the i the cross-validation iteration.

The final model quality is calculated as the average loss across all validation iterations

14 $$L=\frac{1}{k}\sum_{i-1}^{k}L_{i}$$

Due to incomplete data in 2018 and 2024, the 2019–2023 period was selected for time-series cross-validation. This data set spans five years (60 months). For months with missing observations, linear interpolation was used to fill the gaps. The parameters of the TSCV process were set as follows: time-series length *n* = 60, training window *w* = 24, and test window *m* = 6, resulting in k = 6 validation iterations.

#### 2.5.3 Corrosion Scale Analyses

The results provide supporting evidence for understanding the corrosion mechanisms in the purification system. Corrosion-product samples were collected during on-site corrosion investigations in representative high-risk locations. The collected samples were subsequently analyzed in the laboratory using scanning electron microscopy (SEM) to characterize surface morphology and X-ray photoelectron spectroscopy (XPS) to determine the chemical composition of corrosion products.

## 3 Results and Discussion

### 3.1 Corrosion Data Analysis

The overall characteristics of the corrosion monitoring data sets and inspection infrastructure are summarized in Figure 3. As shown in Figure 3a, the temporal distribution of inspection records exhibited noticeable fluctuations over the years, which may be related to adjustments in inspection strategies, changes in inspection budgets, and variations in maintenance planning. The spatial distribution of pipeline components across purification trains and major process units are illustrated in Figure 3b,c. Straight pipes comprised most records, followed by elbows and reducers, with fewer from tees and valves. The desulfurization unit yielded the most monitoring measurements, whereas the dehydration and tail gas recovery units yielded fewer.

**Figure 3:**
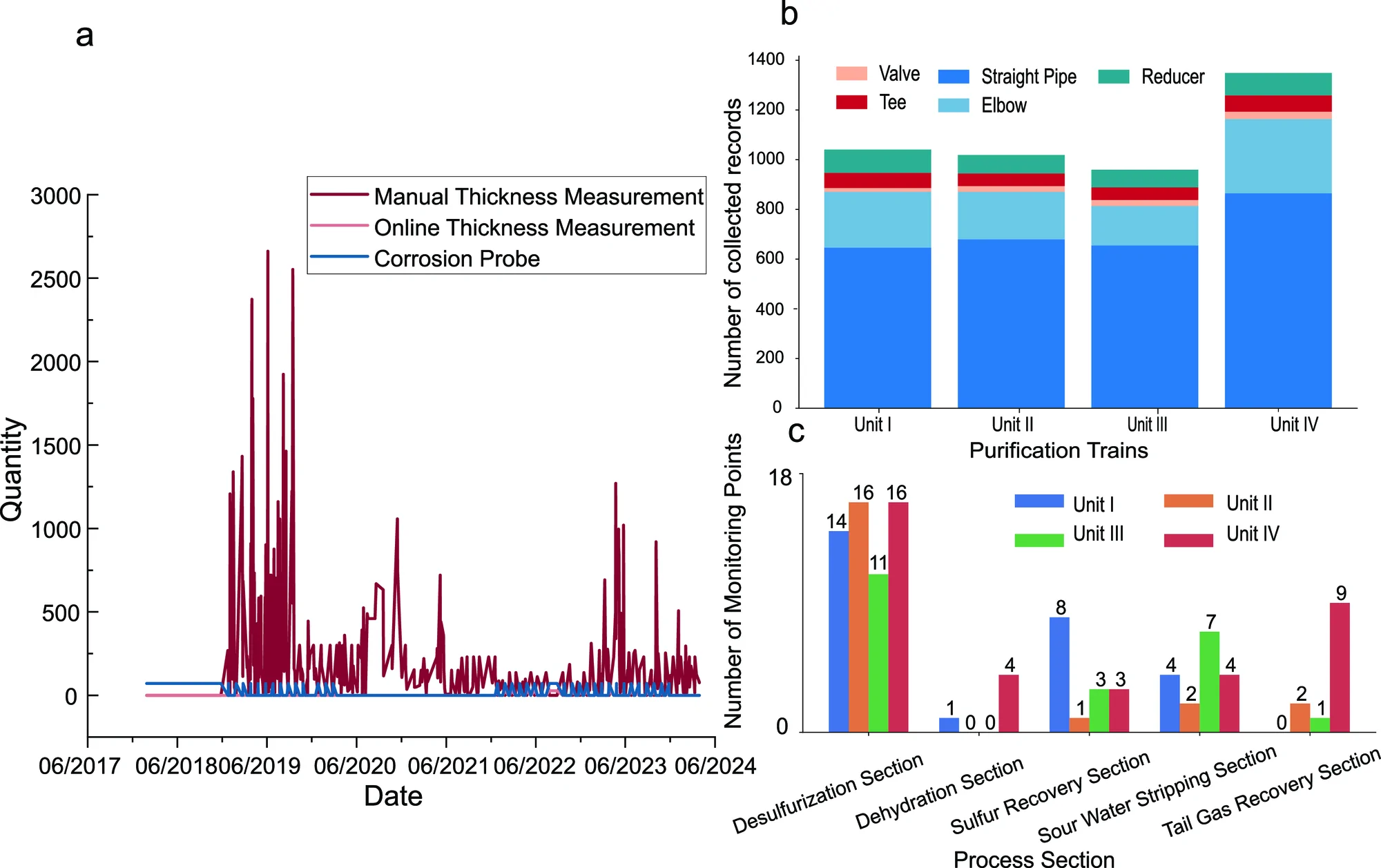
Overview of Corrosion Monitoring Data and Infrastructure in the Natural Gas Purification Plant. (a) Temporal distribution of corrosion monitoring records obtained from manual ultrasonic thickness measurements, online monitoring systems, and corrosion probes from 2017 to 2024. (b) Distribution of pipeline component types across different purification trains. (c) Distribution of corrosion monitoring points across major process units in different purification trains.

Figure 4 shows the uneven distribution of corrosion monitoring density across pipeline loops and processing units. Rinse return, regeneration return, and back gas pipelines had high inspection point counts, whereas fuel gas, circulating water, and venting lines had far fewer. This indicated that monitoring density did not fully align with potential corrosion risk. Some low-risk loops remained heavily monitored, while corrosive environments such as condensate pipelines, pump outlet sections, and certain gas-processing lines remained under-monitored.

**Figure 4:**
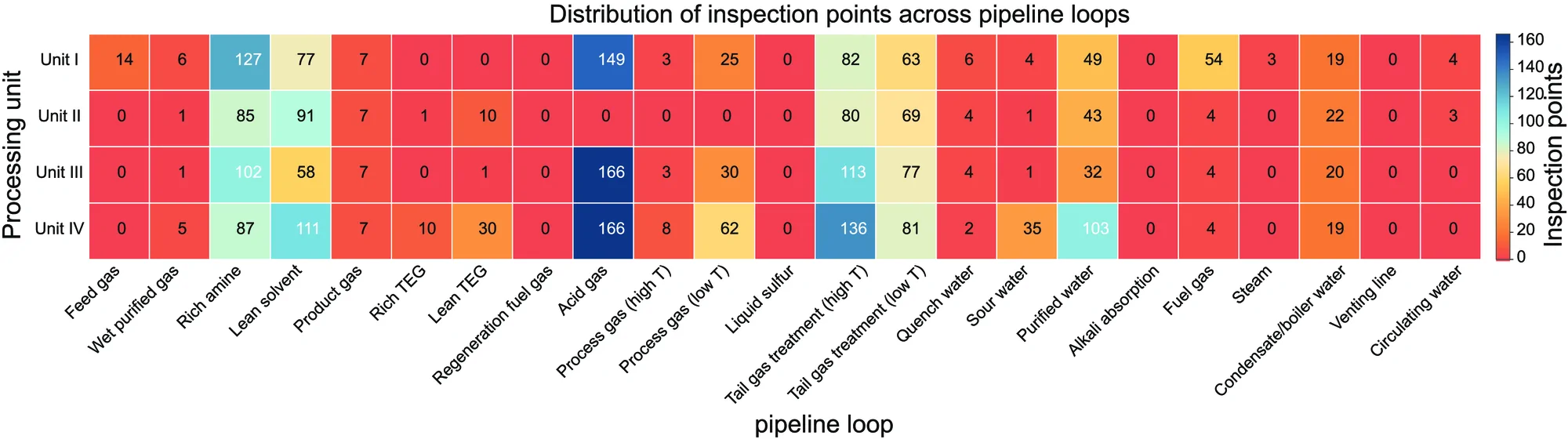
Heatmap showing the distribution of inspection points across process loops in different processing units.

Analysis of the monitoring data indicated that although monitoring had been implemented at some key pipeline sections, inspection records at certain structural hotspots remained limited. For example, several elbows and branch connections showed fewer inspection records, suggesting that not all potential corrosion-sensitive structural locations were fully covered. A previous study showed that structural discontinuities could alter local flow patterns and generate turbulence, potentially enhancing localized or flow-accelerated corrosion.^25^

These results indicated that the corrosion monitoring system in the purification plant exhibited significant spatial heterogeneity. The distribution of inspection points appeared to be influenced more by maintenance strategies and inspection planning than by actual corrosion susceptibility. As a result, several structurally sensitive locations, such as elbows and branch connections where localized corrosion was more likely to occur, remained relatively under-monitored. This uneven inspection coverage limited the ability of the existing monitoring records to represent the overall corrosion risk distribution across the complex pipeline network. Therefore, data-driven analysis and prediction approaches were necessary to complement conventional monitoring practices and help identify potential corrosion-prone regions that might not be adequately covered by the current inspection infrastructure.

### 3.2 Corrosion Data Modeling

Data-driven prediction models rely on sufficient historical observations to ensure model feasibility and reliable predictive performance.^26^ However, the results showed that approximately 60% of the monitoring locations were measured only once. After screening the data set, 269 monitoring locations were identified as having sufficient observations for time-series modeling using the ETS–Theta approach (Table 2), which was considerably higher than the number of eligible locations for other model types. Although only 269 monitoring locations satisfied the requirements for time-series modeling, these locations were distributed across major corrosion-sensitive process environments, including acid gas (AG), acid water (AW), and low-pressure condensate (LC) systems. Therefore, the data set still captured representative corrosion behaviors under different operating conditions in the purification plant.

**Table 2:** Comparison of Modeling Eligibility for Different Prediction Models

model name	eligible locations	theoretical basis	advantages	limitations
ARIMA	10	linear time series	simple structure	requires stationarity
SVR	31	function regression	handles nonlinear data	sensitive to parameters
LSTM	26	RNN-based model	captures long-term patterns	data-intensive
ETS and Theta	269	trend decomposition	strong trend extraction	weak for sudden changes

Nevertheless, inspection coverage across the plant was uneven and partially influenced by maintenance strategies and inspection planning. Consequently, the identified corrosion hotspots may also reflect existing inspection bias to some extent. These findings indicate that the current monitoring infrastructure cannot fully represent the overall corrosion risk distribution of the entire plant and should be further improved through optimized inspection allocation and enhanced data acquisition.

Among the 269 monitoring locations suitable for time-series modeling, the hybrid ETS–Theta model identified several as high-risk corrosion points due to relatively rapid predicted thickness reduction trends (Figure 5). Most of these were distributed in acid gas (AG) pipelines and low-pressure condensate (LC) systems, suggesting that corrosion risk was concentrated in sour gas service. This pattern is consistent with previous studies showing that exposure to CO_2_ and H_2_S containing streams significantly increases the susceptibility of pipeline steels to internal corrosion.^27^

**Figure 5:**
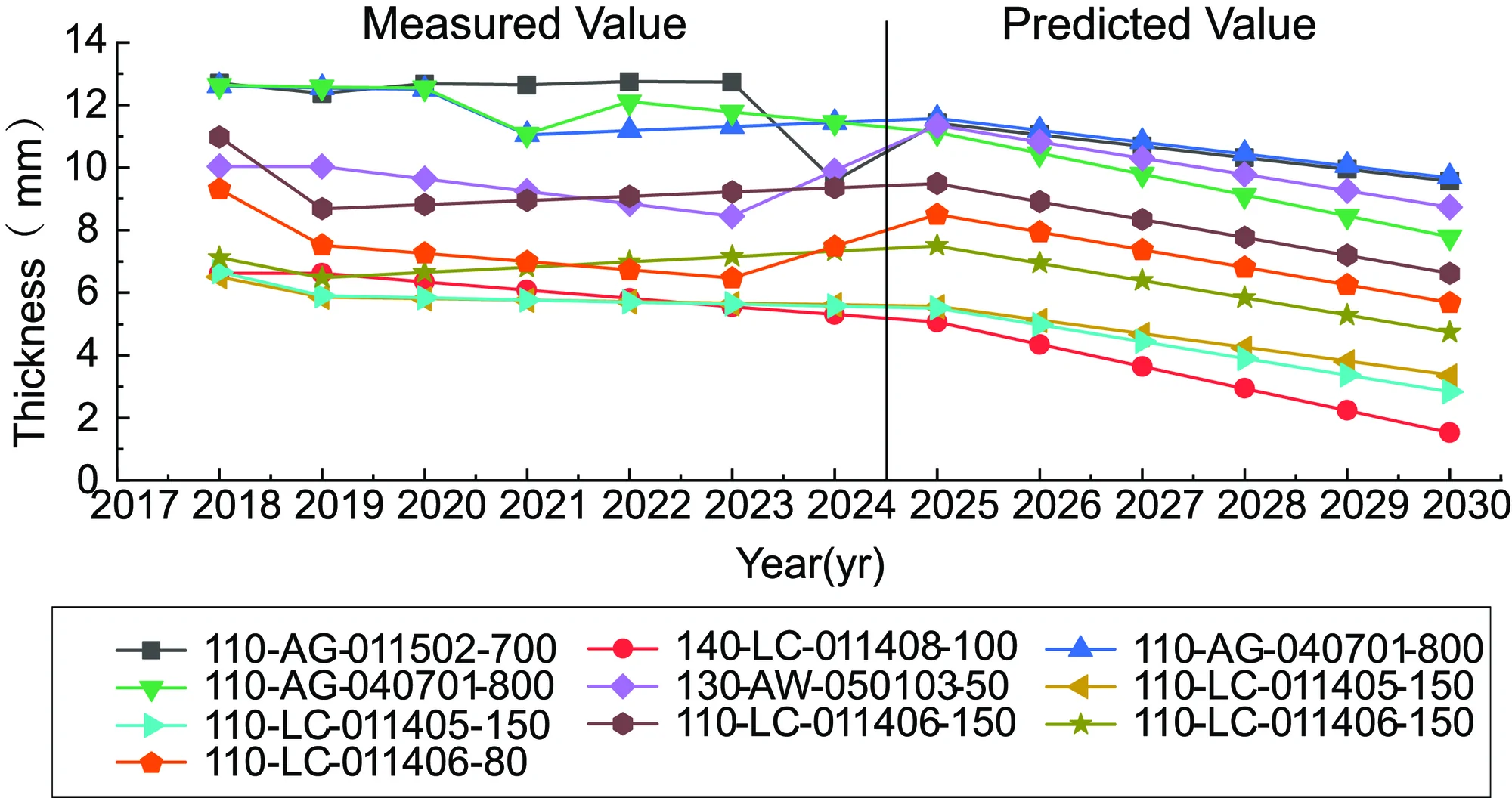
Key Point Prediction Results Chart of ETS-Theta Combined Model.

The spatial distribution of predicted high-risk regions aligned with engineering observations in natural gas purification systems. AG and acid water (AW) process units were commonly considered corrosion-prone due to the presence of acidic gases and corrosive aqueous phases.^28,29^ Additionally, several high-risk locations in LC pipelines were associated with condensate-induced corrosion. In these pipelines, a decrease in pipe wall temperature could lead to water condensation and the formation of an electrolyte film; once H_2_S and CO_2_ dissolved into the condensate, localized corrosion on the pipe surface could be accelerated.^30,31^

### 3.3 Model Performance Evaluation

Table 3 summarizes the quantitative evaluation of model prediction performance. The results show that the ETS–Theta hybrid model achieved the best overall performance, with an *R* ^2^ value of 0.853, together with the lowest MAE (0.13) and lowest sMAPE (9.1) among the tested models, indicating improved prediction accuracy and stability in corrosion thickness forecasting.

**Table 3:** Evaluation Metrics of Model Prediction Results

metric	ETS model	theta Mode	hybrid model
*R* ^2^	0.76	0.58	0.853
sMAPE	14.7	14.7	9.1
MAE	0.27	0.32	0.13

### 3.4 Semi-Empirical Model Comparison and Time-Series Validation

To further evaluate the reliability of the time-series modeling results, semiempirical corrosion models including Norsok and de Waard–Milliams (DWM) were applied for comparison in a high-sulfur operating environment. The results show that the hybrid ETS–Theta model achieved substantially better fitting performance than the individual ETS and Theta models when compared with semiempirical predictions (Figure 6).

**Figure 6:**
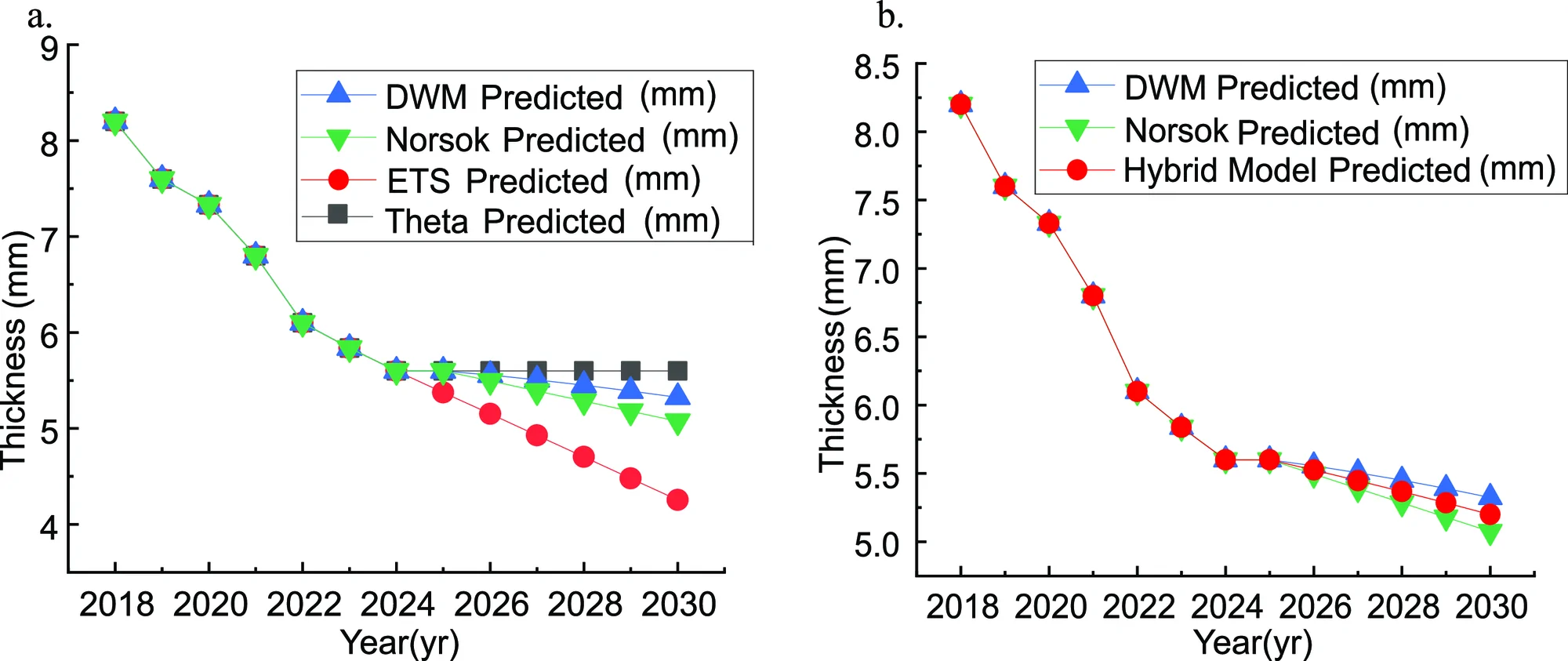
Fit plots of time-series models and semiempirical models:((a) representative fitting result for a corrosion monitoring location; (b) representative fitting result for another corrosion monitoring location. The ETS–Theta hybrid model generally exhibited closer agreement with the observed thickness-loss trend than the individual models.).

The predictive performance of the hybrid model was further evaluated using rolling time-series cross-validation. The results indicate that the hybrid model achieved an average prediction performance of 0.155 mm/a (MAE) and 11.53% (sMAPE) (Table 4). Moreover, as the size of the training data set increased, both MAE and sMAPE gradually decreased, suggesting that model accuracy improves as additional monitoring data become available.

**Table 4:** TSCV Validation Results (*n* = 269)

validation round	training set range	test set range	MAE(mm/a)	sMAPE(%)
1	2019–2020.12	2021.1–2021.6	0.20	13.3
2	2019–2021.6	2021.7–2021.12	0.18	12.6
3	2019–2021.12	2022.1–2022.6	0.17	11.9
4	2019–2022.6	2022.7–2022.12	0.15	11.1
5	2019–2022.12	2023.1–2023.6	0.12	10.8
6	2019–2023.6	2023.7–2023.12	0.10	9.5

Multiparameter machine learning models have been reported to improve corrosion prediction accuracy when sufficient environmental parameters are available. For example, Dong et al.^32^ developed Random Forest and Support Vector Regression models using multiple input variables to predict corrosion current density. The time-series models rely mainly on historical monitoring data and therefore require fewer types of input parameters. Similar observations have been reported in studies using industrial inspection data sets, where time-series forecasting models demonstrate reliable predictive performance when long-term monitoring records are available.^33^

The improved performance of the hybrid ETS–Theta model was attributed to the complementary characteristics of its two components. Previous studies have shown that combining multiple forecasting models enhances prediction robustness and reduces model-specific biases in complex temporal data sets.^34^ The ETS model captured the level and trend components of time series through exponential smoothing, while the Theta method decomposed the original series into modified trend components to better represent long-term trends and short-term fluctuations.^35,36^ By integrating these two approaches, the hybrid model captured both short-term variability and long-term corrosion evolution patterns. Rolling validation results further demonstrated that prediction accuracy remained relatively stable as the training and testing windows moved forward along the time axis, which indicated good robustness of the time-series modeling framework under practical operating conditions.

Kindly confirm in your author response whether the photos in Figure 7 were taken by one of the authors. In case it was not, please clarify if permission is required. If permission is required, provide a document that shows a granted permission, and add a credit line accordingly.

**Figure 7:**
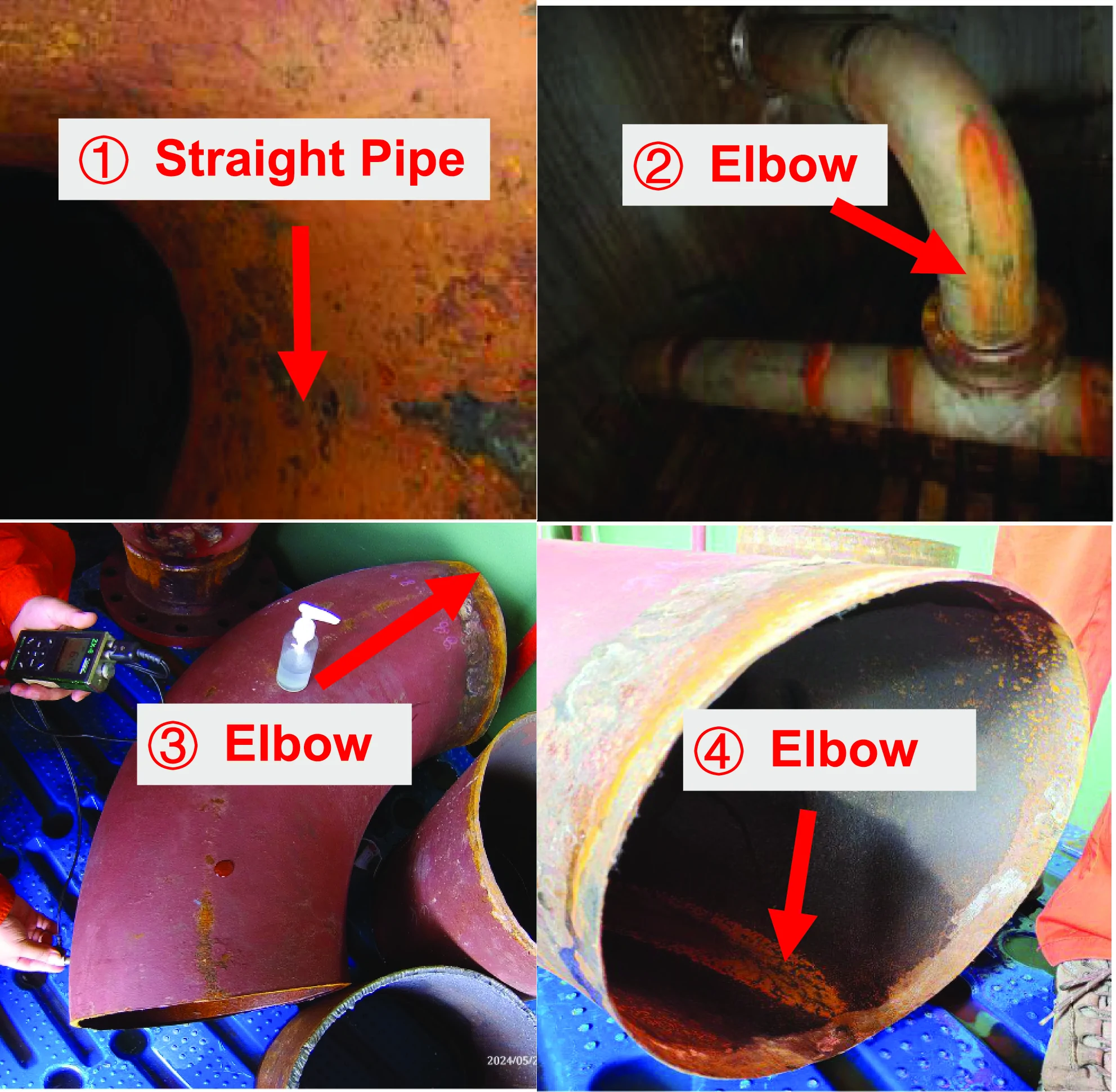
Corrosion Demonstration from Field Investigation Consistent with Model-Predicted Trends in High-Risk Areas.

### 3.5 On-Site Corrosion Investigation and Mechanism Analysis

To further interpret the physical mechanisms underlying the model-predicted corrosion hotspots, the major process loops were categorized according to operating conditions, corrosive media, dominant corrosion mechanisms, failure modes, and average corrosion rates, as summarized in Table 5. Different process environments exhibited distinct corrosion characteristics, providing mechanistic support for the spatial distribution of high-risk regions identified by the ETS–Theta model.

**Table 5:** Corrosion Characteristics and Dominant Corrosion Mechanisms of Major Process Loops in a High-Sulfur Natural Gas Purification Plant

process loop	corrosive medium	temperature (°C)	pressure (MPa)	average corrosion rate (mm/a)	dominant corrosion mechanism	failure mode
Acid Gas (AG) transportation system	H_2_S- and CO_2_-containing sour gas	40	0.1–5.8	0.498	wet H_2_S corrosion; CO_2_ corrosion	uniform wall thinning of pipelines
Acid Water (AW) treatment system	H_2_S/CO_2_-containing acidic water	65	0.5	0.521	acid water corrosion; pitting corrosion	pipeline wall thinning; perforation
low-pressure condensate system	recirculating cooling water	48	0.1–0.3	0.535	oxygen corrosion; microbiologically influenced corrosion (MIC)	pitting corrosion

Among the identified hotspots, AG, AW, and LC systems exhibited elevated corrosion risk due to the combined effects of H_2_S, CO_2_, acidic water, and condensate formation. Corrosion in these systems was primarily driven by electrochemical reactions, localized attack, and under-deposit corrosion. In addition, structural features such as elbows, reducers, and welded joints promoted local turbulence, condensate accumulation, and mass-transfer enhancement, which accelerated wall thinning and pitting corrosion. These findings indicate that the observed corrosion hotspots resulted from the combined influence of corrosive media, operating conditions, and flow-related factors rather than sour-service conditions alone.

Field observations generally agreed with model trends. As shown in Figure 7, high-risk monitoring points exhibited corrosion thinning, localized pitting, and under-scale corrosion, particularly at pump outlet reducers, elbows, and welded joints. These observations are consistent with previous studies indicating that corrosion in sour-gas environments tends to concentrate at structurally sensitive locations. In addition to sour-service conditions, local turbulence, flow acceleration, condensate accumulation, and gas–liquid alternation enhanced corrosive species transport and promoted localized corrosion^37^ These coupled effects provide a mechanistic explanation for the corrosion hotspots identified by the ETS–Theta model.

Figure 8 shows the morphology and composition of corrosion products on the inner pipe wall. SEM images (Figure 8a,b) reveal a porous, agglomerated structure with irregular clusters and layered particles. Fine particles deposited on larger products indicate continuous growth. Such porous structures may facilitate corrosive species penetration and promote localized corrosion. XPS spectra (Figure 8c,d) identify metal carbonates, oxides, Fe_3_O_4_, Fe_2_O_3_, and FeO, suggesting combined oxidation and acidic gas corrosion, consistent with sour gas purification conditions.

**Figure 8:**
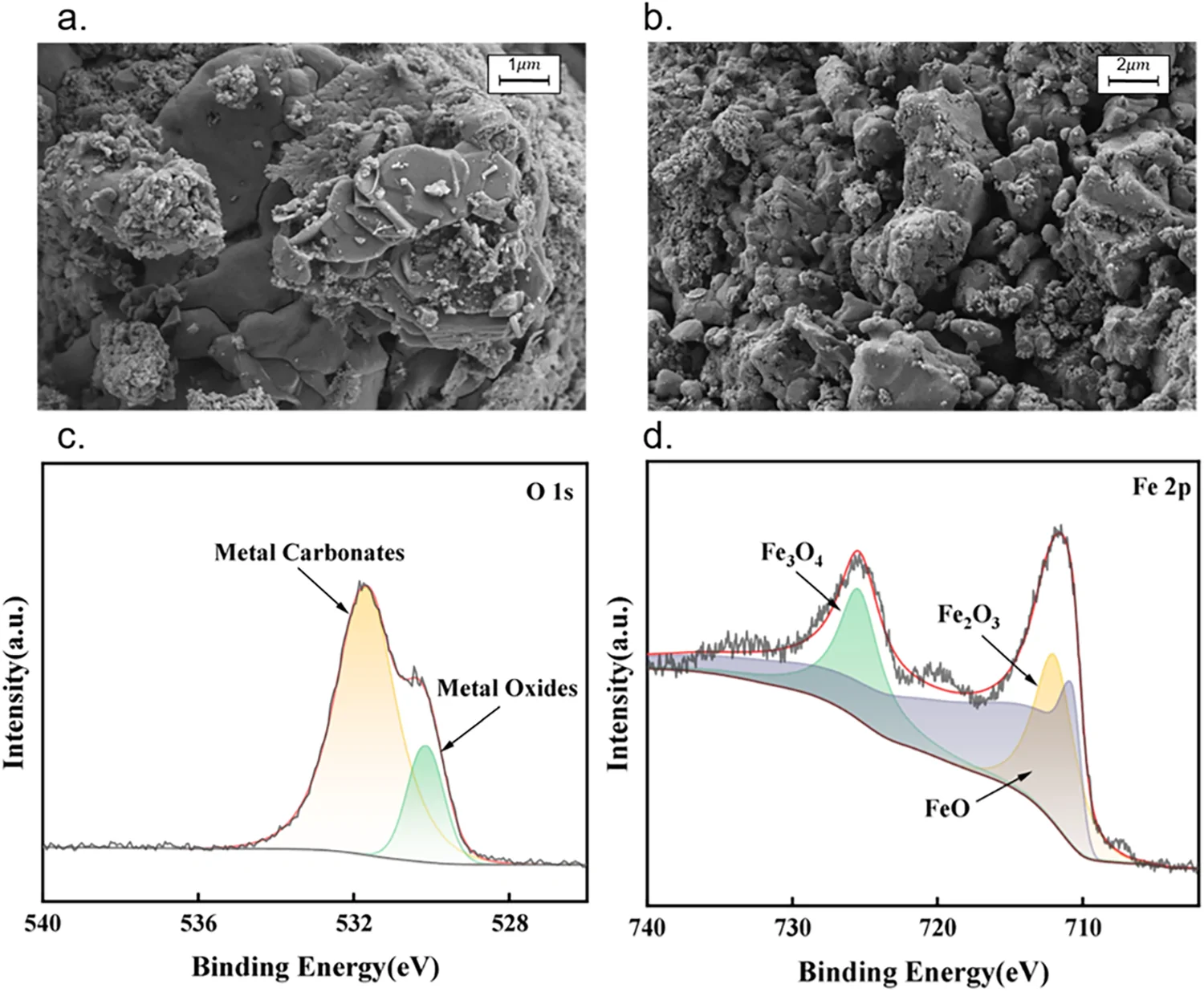
Characterization of corrosion products on the inner pipe wall: (a, b) SEM images showing porous and agglomerated corrosion-product morphology; (c) O 1s XPS spectrum indicating the presence of metal carbonates and metal oxides; (d) Fe 2p XPS spectrum showing Fe_3_O_4_, Fe_2_O_3_, and FeO species.

The SEM and XPS results further support the mechanism interpretation proposed above. The porous corrosion-product structure facilitates the penetration of corrosive species, while the coexistence of iron oxides and carbonate-containing corrosion products indicates the combined influence of oxidation, CO_2_ corrosion, and H_2_S-related corrosion processes. These findings suggest that corrosion occurrence in high-sulfur natural gas purification systems is governed by the interaction of corrosive media, operating conditions, flow disturbance, and corrosion-product stability rather than a single corrosion mechanism.

Together with a previous study, these mechanistic analyses indicate that corrosion behavior in such systems is governed by multiple coupled factors, making the corrosion mechanisms complex and potentially leading to discrepancies between predicted and observed severity.^38^

### 3.6 Significance and Limitations

This study demonstrates that organizing long-term corrosion monitoring data into a unified database enables more systematic analysis of corrosion evolution in complex purification systems. By integrating monitoring data sets with data-driven analytical methods, potential and previously underemphasized corrosion-prone regions (e.g., LC systems) can be identified precisely and quantitatively. As illustrated in Figure 9, the corrosion database developed in this study can serve as the foundation for a broader multiplant corrosion management framework. By integrating monitoring data from different purification plants into a unified database, data-driven models can be continuously optimized to support weak-area identification, corrosion risk prediction, and deep hidden-information mining, while feedback from model outputs can further guide data collection and database improvement.

**Figure 9:**
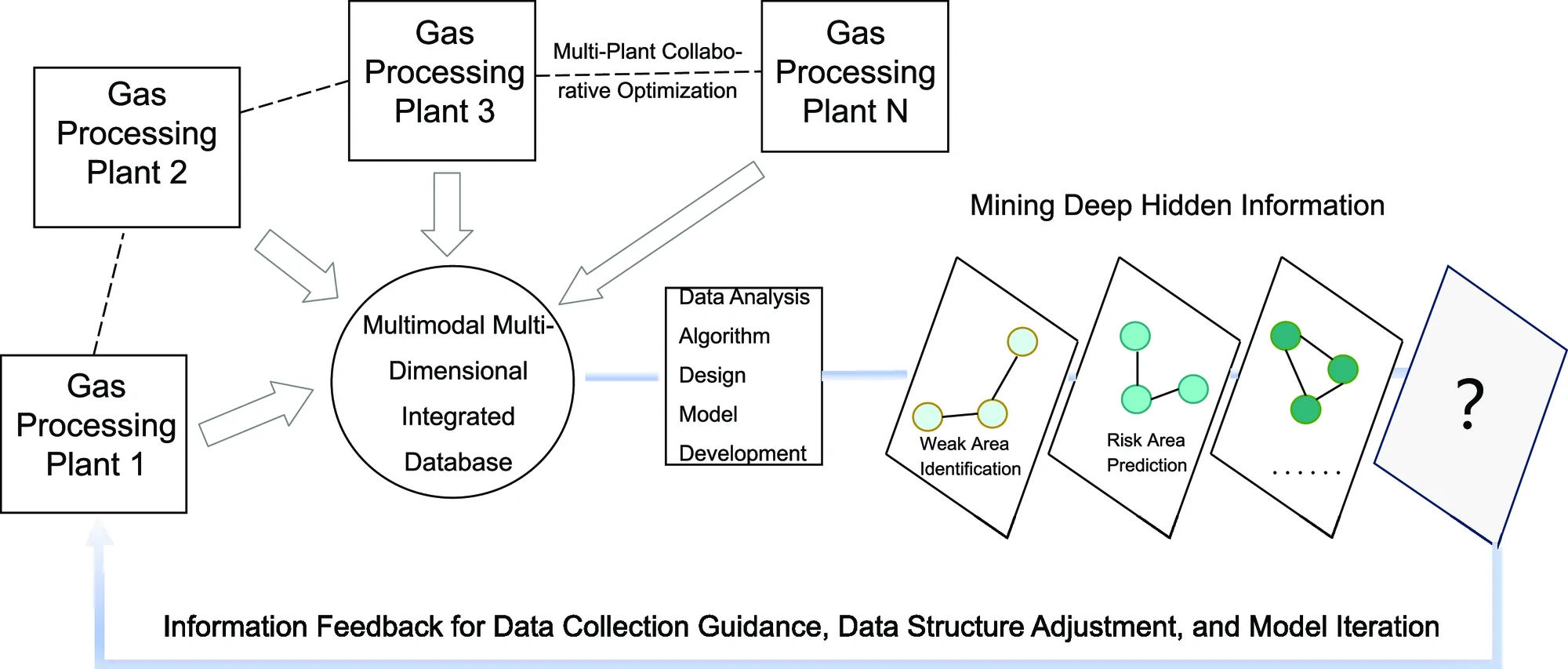
Conceptual Framework for Multi-Plant Spatiotemporal Corrosion Prediction and Deep Identification.

In addition to identifying corrosion-prone regions, the predicted wall-thickness evolution trends can support maintenance planning and inspection optimization. Monitoring locations exhibiting rapid thickness loss may be prioritized for more frequent inspection and remaining-life assessment, whereas locations with relatively stable trends may adopt extended inspection intervals.

Unlike previous studies that focused primarily on laboratory data sets or short-term monitoring records, this study established a unified corrosion database containing more than 100,000 inspection records and integrated large-scale data governance, anomaly detection, time-series forecasting, semiempirical validation, and field investigation within a single analytical framework. Nevertheless, despite the large number of inspection records, the available data remain insufficient for comprehensive corrosion risk assessment. Many monitoring locations were inspected only once or at irregular intervals, which limits accurate characterization of long-term corrosion trends. These findings indicate that corrosion monitoring systems in purification plants should be further improved through enhanced data acquisition, standardized inspection procedures, and systematic data management. While the conclusions are not yet robust, this work offers a potentially valuable framework for future studies.

## 4 Conclusions

In this study, over 100,000 corrosion monitoring records from a high-sulfur natural gas purification plant were compiled and analyzed. Based on these operational data, a preliminary time-series prediction framework was explored to investigate the temporal evolution and spatial distribution of corrosion risk in complex purification systems. The main conclusions are as follows:

(1) The results showed that the data-driven approach had the potential to be implemented in high-sulfur natural gas purification plants. Large-scale corrosion data analyses also revealed that several potential corrosion locations were under-monitored.
(2) The ETS and Theta time-series models demonstrated reasonable adaptability to field corrosion monitoring data. The ETS–Theta ensemble model exhibited relatively stable prediction performance under the current data conditions.
(3) The predicted spatial distribution indicated that acid gas (AG), acid water (AW), and low-pressure condensate (LC) systems remained major corrosion-prone regions, which was generally consistent with field observations and engineering experience.
(4) Owing to the limitations found in the current monitoring data sets, modeling results should be cautiously interpreted, highlighting the need for improved corrosion monitoring strategies and data management.
